# Dietary Vitamin B6 Deficiency Impairs Gut Microbiota and Host and Microbial Metabolites in Rats

**DOI:** 10.3390/biomedicines8110469

**Published:** 2020-11-02

**Authors:** Shyamchand Mayengbam, Faye Chleilat, Raylene A. Reimer

**Affiliations:** 1 Department of Biochemistry, Memorial University of Newfoundland, St. John’s, NL A1C 5S7, Canada; smayengbam@mun.ca; 2 Faculty of Kinesiology, University of Calgary, Calgary, AB T2N 1N4, Canada; fatima.chleilat1@ucalgary.ca; 3 Department of Biochemistry and Molecular Biology, Cumming School of Medicine, University of Calgary, Calgary, AB T2N 4N1, Canada

**Keywords:** vitamin B6, pyridoxal, gut microbiota, metabolomics, sex effect

## Abstract

Vitamin B6 plays a crucial role as a cofactor in various enzymatic reactions but bacteria-produced vitamin B6 is not sufficient to meet host requirements. Our objective was to assess the impact of diet-derived vitamin B6 on gut microbiota and host serum metabolomics. Sprague–Dawley rats (*n* = 47) were fed a control, low B6 (LB6) or high B6 (HB6) diet for six weeks. Serum and cecal samples were collected for biochemical, metabolomics and gut microbiota profiling. There was a significant sex effect for gut microbiota and several metabolic markers. Bodyweight and percent body fat were significantly reduced in LB6 compared to control and HB6 rats. Microbial beta-diversity differed significantly between LB6 and the control and HB6 rats in both sexes. *Lachnospiraceae*_NK4A136_group and *Bacteroides* were the primary taxa driving the difference between LB6 and control. There was a significant separation of cecal and serum metabolites of LB6 compared to control and HB6 rats. In the cecum, arginine biosynthesis was impaired, while vitamin B6 metabolism, lysine degradation and nicotinate and nicotinamide metabolism were impaired in serum metabolite profiles. Cecal propionate and butyrate were significantly reduced in LB6 rats irrespective of sex. Host vitamin B6 deficiency but not excess significantly alters gut microbial composition and its metabolites.

## 1. Introduction

Micronutrients, especially vitamins, play a crucial role in several metabolic reactions [1]. The deficiency of such nutrients is observed in both underdeveloped and developed societies [2]. Although the role of dietary factors in modulating gut microbiota composition and their influence on the host physiology are well documented [3], little is known about how micronutrient deficiencies affect gut microbial ecology. At least 100 bacterial species can synthesize B-vitamins, cooperatively exchanging them to ensure survival [4]. Different species may encode different biosynthetic pathways, while other taxa have complementary functions related to the vitamins [5]. For instance, Bacteroidetes possess a canonical thiamin biosynthesis pathway and the organisms that lack this pathway, such as *Salmonella enterica*, depend on the presence of transporters to acquire thiamin [6]. Using systematic genome assessment, Magnusdottir et al. identified riboflavin and niacin as the two most commonly synthesized B-vitamins in the gut [4]. According to their estimation, gut microbiota could not provide enough B-vitamins to meet the host’s daily requirement. Additionally, a large portion of the bacterially synthesized vitamins must compete between the host and the non-producers in the gut [4]. As a result, the basic needs of such micronutrients largely depend on external supply through the diet. Recently, Hibberd et al. examined the effect of vitamin A, folate, iron and zinc deficiencies on bacterial species from human gut microbiota using a gnotobiotic mouse model [7]. Interestingly, acute vitamin A deficiency had the most substantial effect on the bacterial community structure, and *Bacteroides vulgatus* was identified as the prominent responder [7].

Vitamin B6 is one of the vital micronutrients involved in one-carbon metabolism along with folate and vitamin B12. Pyridoxal 5-phosphate (PLP), the active form of vitamin B6, acts as a cofactor in more than 140 enzymatic reactions in carbohydrate, amino acids and lipid metabolism [8]. It has also been shown to have antioxidant [9] and anti-inflammatory properties [10]. Recently, Walden et al. demonstrated its novel role in the immune response by regulating the interaction between the BRISC-SHMT2 complex [11]. Due to the multifaceted roles of vitamin B6, it is expected to have a significant impairment in metabolic pathways if its concentration deviates substantially from optimal levels. Notably, the prevalence of vitamin B6 deficiency is as high as 12.4% in healthy young adult women in Vancouver, Canada [12], and up to 32% in the female US population [13].

The gut microbiota have co-evolved in the mammalian gut, and their biosynthetic capacity depends in large part on the host environment. Therefore, it is plausible that micronutrients, including vitamin B6, could play a critical role in shaping microbiota composition and metabolic capacity. In bacteria such as *Escherichia coli*, vitamin B6 is synthesized in the form of PLP from different precursors including, deoxyxylulose 5-phosphate, 4-phosphohydroxy-L-threonine, glyceraldehyde-3-phosphate and D-ribulose 5-phosphate [8]. Therefore, the efficiency of such de novo biosynthesis of vitamin B6 also depends on the substrates’ availability in the gut. In the current study, we investigated the influence of dietary vitamin B6 on gut microbiota composition and host and microbial metabolites using a rat model of a pyridoxine deficiency or surplus.

## 2. Materials and Methods

### 2.1. Animals and Diet

Weanling Sprague–Dawley rats (3 weeks of age; male *n* = 24, female *n* = 23) were obtained from the University of Calgary Animal Care facility (Calgary, Canada). Animals were housed two to three rats per cage in a humidity-controlled room maintained at 22 °C with a 12h reverse light-dark cycle. After a 1 week acclimatization period with a standard chow diet, rats were fed an AIN-93G-based diet with optimum vitamin B6 (Control, 7 mg/kg of pyridoxal.HCl), low vitamin B6 (LB6, 0.07 mg/kg pyridoxal.HCl) and high vitamin B6 (HB6, 70 mg/kg pyridoxal.HCl) levels for 6 weeks. Diets were purchased from Dyets Inc. (Bethlehem, PA, USA). Rats had access to food and water ad libitum. Food intake was measured every other day, while body weight was measured weekly. Following the 6 week feeding period, rats were lightly anesthetized with isoflurane and scanned using dual x-ray absorptiometry (DXA) (Hologic ODR 4500; Hologic Inc., Marlborough, MA, USA) for lean and fat mass, bone mineral content and density using Hologic QDR software for small animals. Rats were then fasted overnight for 12 h and euthanized by overanesthetization and aortic cut. Blood and cecal matter were collected and stored at −80 °C until further analysis. Ethical approval was granted by the University of Calgary Animal Care Committee (#AC18-0074; December 18, 2018) and followed the Canadian Council on Animal Care guidelines.

### 2.2. Biochemical Markers

Serum glucose (Glucose Liquicolor test, Stanbio Laboratory, Boerne, Texas, USA), insulin (Rat/mouse insulin ELISA kit, Millipore Sigma, Burlington, Ontario, Canada) and triglycerides (Triglycerides Liquicolor test, Stanbio Laboratory, Boerne, Texas, US) were measured using commercially available ELISA-based kits following the manufactures instructions. Homeostatic Model Assessment of Insulin Resistance (HOMA) was calculated based on the standard formula (HOMA-IR = (fasting serum insulin (μU/mL)*fasting serum glucose (mmol/L)/22.5).

### 2.3. Gut Microbiota Sequencing

Cecal genomic DNA was extracted using a FastDNA spin kit with a bead-beating step (MP Biomedicals, Lachine, QC, Canada) and the microbial composition determined using Ilumina’s 16S rRNA gene sequencing protocol (Illumina, San Diego, CA, USA) of the V3-V4 regions as previously described [14]. Sequencing primers and low-quality reads were removed using Trimmonatic version 0.39 [15]. A table of amplicon sequence variants (ASVs) was generated using DADA2 version 1.12 [16], and taxonomic classification was done using Silva v138 database. Raw sequence data are available upon request.

### 2.4. Metabolomics Analysis

Metabolomics analysis was performed with liquid chromatography-Quadrupole time of flight-mass spectrometry (LC-QTOF-MS) using an untargeted approach. For gut metabolomics, cecal samples (200 mg) were extracted with 1mL ice-cold MS-grade water. The supernatant underwent a freeze and thaw cycle followed by centrifugation at 14,000 rpm for 15 min for 7-times. The supernatant was filtered through a 0.2 µm filter before injecting it into the LC-QTOF-MS [17]. For serum metabolomics, serum samples (50 µL) were extracted with 200 µL ice-cold MS-grade methanol. After centrifugation, the supernatants were dried in a SpeedVac at 30 °C. The dried samples were reconstituted in 50% methanol before injecting it into the LC-QTOF-MS [18]. Chromatographic separations were performed on an Acquity HSS T3 column (2.1 × 1.8 μm, Waters, Mississauga, ON, Canada) using a gradient programming of MS-grade water and acetonitrile containing 0.1% formic acid. The eluted metabolites were analyzed in positive electrospray ionization mode with a full-scan ranging from 50 to 1000 m/z [18]. All the raw files were first converted to mzML files by ProteoWizard version 3.0 (Pala Alto, CA, USA), and data were processed for peak detection, retention time correction, alignment and molecular features annotation using XCMS online version 3.7.1 [19].

### 2.5. Short-Chain Fatty Acid Analysis

Short-chain fatty acids (SCFA) were extracted from cecal samples (150 mg) using acidified water and derivatization as previously described [20] with few modifications. Briefly, 100 uL of the extract was derivatized in 3-nitrophenylhydrazine hydrochloride in pyridine medium. SCFA- hydrazides were dissolved in diethyl ether. After evaporation, the SCFAs were dissolved in 50% methanol and injected into the high-performance liquid chromatography-diode array detector (HPLC-DAD). The analytes were separated with the use of C18 column (150 mm, 4.6 um, 2.6 um) at 40 °C using a gradient solvent of water and acetonitrile. SCFA-hydrazides were detected at the wavelength of 230 nm.

### 2.6. Statistical Analysis

All statistical analyses were performed in GraphPad Prism (version 6; San Diego, CA, USA) and R, version 4.0.0. Biochemical outcomes with a single time point (e.g., body fat, glucose, etc.) were analyzed with a two-way ANOVA to determine the effects of diet and sex and their interaction. If the sex effect was identified, male and female data were analyzed separately. Tukey’s post-hoc test was used to determine group differences. Microbial data were analyzed using the DADA2 pipeline [16] and the DESeq package [21]. We used the Silva database for the taxonomic assignments. Logarithmic transformation, centering and scaling were performed before conducting statistical analysis if needed. We examined alpha and beta-diversity to study the richness and composition of the gut bacteria. The Wilcoxon test was undertaken to identify the significant taxa between two treatment groups at fdr *p* value ≤ 0.05. For metabolomics analysis, partial least square discriminant analysis (PLS-DA), was performed using the ropls package as described previously [22]. The model’s quality was assessed using R2Y and Q2Y with the sevenfold cross-validation method at *p* < 0.05. In order to identify the metabolic pathways that differed between the treatment groups, pathway analyses were performed with MetaboAnalyst 4.0 (Montreal, QC, Canada) by following the Mummichog algorithm-based MS peak to pathway analysis procedure [23]. Metabolic pathways with -log *p*-value > 1 were considered significantly enriched. A correlation analysis between the significant microbial taxa with clinical markers and putatively identified metabolites was also performed using the corrplot package in R.

## 3. Results

### 3.1. Physical and Clinical Markers

**Figure 1:**
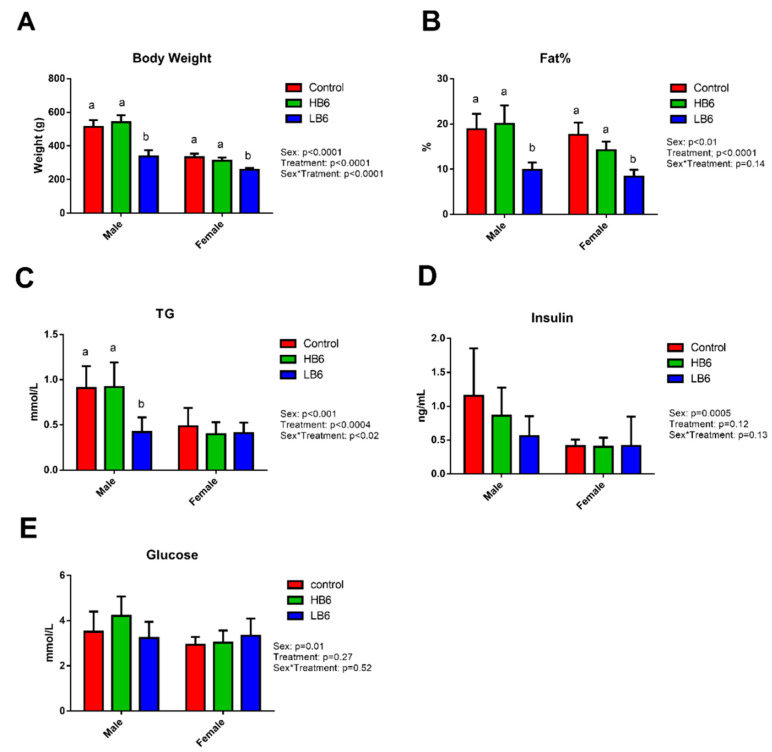
Primary physical and clinical markers showing effects of low and high dietary vitamin B6 levels on body weight (**A**), percentage fat (**B**), triglycerides (**C**), insulin (**D**), and glucose (**E**) in rats. Data represent mean ± SD (*n* = 7–8/group of each sex). Values with different superscripts indicate significant differences between the treatment groups within the same sex (*p* < 0.05).

There was a significant effect of sex on body weight (*p* < 0.0001), percent fat mass (*p* = 0.01) and other clinical markers including, serum triglycerides (TG, *p* < 0.0001), glucose (*p* < 0.01) and insulin (*p* < 0.0005), see Figure 1. Given the significant sex effect, subsequent analyses were performed in males and females separately.

As expected, vitamin B6 deficiency significantly reduced body weight in males and females compared to control and HB6 (Figure 1A). Food intake was also significantly lower in LB6 compared to control and HB6 from week 3 to 6 (Supplementary Figure S1). Compared to control and HB6 rats, LB6 rats had significantly lower percent body fat in males (*p* = 0.0016) and females (*p* = 0.0001) (Figure 1B), serum TG in males (*p* = 0.0003) (Figure 1D), bone mineral content in males (*p* = 0.0001) and females (*p* = 0.0003) and fat and lean mass (*p* < 0.05 for all) (Supplementary Table S1). On the other hand, there was no significant effect of low vitamin B6 on serum glucose (Figure 1E), insulin (Figure 1F) and HOMA-IR (Supplementary Table S1). HB6 did not differ from control in any physical or biochemical markers with the exception that fat mass was significantly lower in females fed HB6 compared to the control (*p* < 0.0001; Figure 1, Supplementary Table S1).

### 3.2. Vitamin B6 Deficiency Altered Gut Microbial Profile in Both Sexes

There was a significant sex effect on the richness and evenness (Shannon index, *p* = 0.009) and between group compositional differences (beta diversity, *p* = 0.038) in the gut microbiota (Supplementary Figure S2A,B). Therefore, analysis of potential treatment effects was performed in males and females separately.

**Figure 2:**
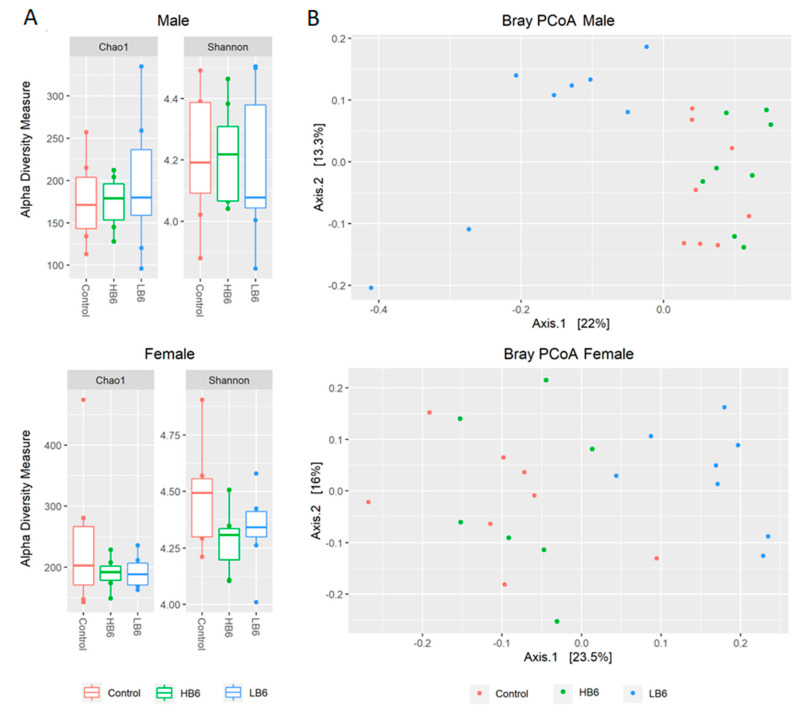
Changes in gut bacteria community composition due to variable levels of vitamin B6 expressed as alpha-diversity (**A**) and beta-diversity (**B**). Alpha-diversity was not significant (Kruskal–Wallis test for α-diversity for male rats (Chao1: *p* = 0.72, Shannon: *p* = 0.88) and female rats (Chao1: *p* = 0.77, Shannon: *p* = 0.20); while beta-diversity according to the Bray–Curtis model was significant (*p* < 0.001 for both sexes), *n* = 7–8/group of each sex).

**Figure 3:**
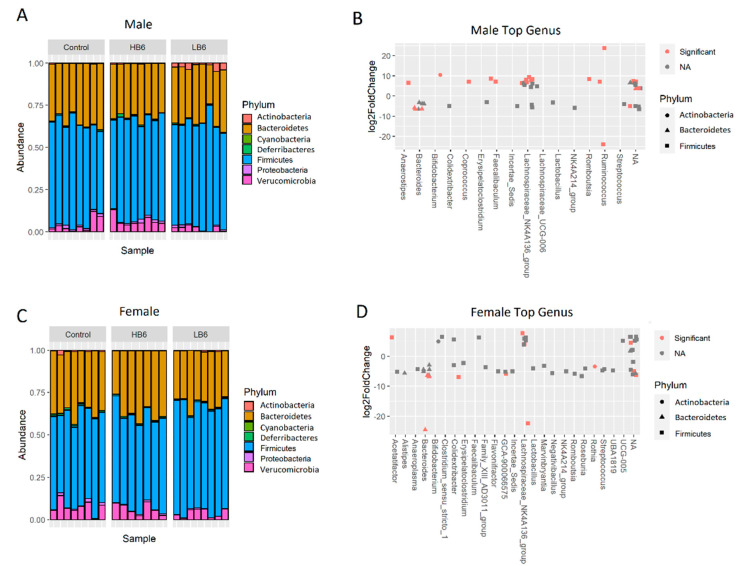
Relative abundance of bacterial taxa using 16S rRNA gene sequencing. Phylum level relative abundance of cecal bacteria for male (**A**) and female (**C**) rats, and the top significantly different genera between the Control and LB6 male (**B**) and female (**D**) rats with their corresponding log2Fold changes. Genera depicted in red are significant at fdr *p*-value < 0.05. *n* = 7–8/group of each sex.

Vitamin B6 deficiency did not alter the species richness in cecal contents in male and female rats analyzed by Chao1 and Simpson indices of alpha diversity (Figure 2). However, beta diversity based on the Bray–Curtis Principal Coordinate Analysis (PCoA) indicated a significant separation of gut microbiota of LB6 rats from the control and HB6 in both male (*p* = 0.001) and female rats (*p* = 0.001). There was no separation between control and HB6 irrespective of sex (male: *p* = 0.17, female: *p* = 0.19) suggesting that vitamin B6 deficiency, but not surplus, altered the composition of the gut microbiota. At the phylum level, we found a lower relative abundance of Verrucomicrobia in LB6 than the control and the HB6 rats (Figure 3A,C). To identify the taxa involved in altering the microbial community, we conducted a Wilcoxon test between the control and the LB6 rats. Supplementary Figure S3 shows the relative abundance of the top taxa at the family level. Here, the relative abundance of *Lachnospiraceae*, *Bacteroidaceae* and *Erysipelatoclostridiaceae* was significantly different in LB6 compared to the control in both sexes. While *Ruminococcaceae*, *Bifidobacteriaceae*, *Peptostreptococcaceae* and *Muribaculaceae* were specific to male rats and *Rikenellaceae* and *Oscillospiraceae* were specific to female rats in response to low B6 levels compared to the control. Similarly, the significant taxa at the genus level are displayed in Figure 3 and show that *Bacteroides* and *Lachnospiraceae*_NK4A136_group were significantly changed in both male and female LB6 rats compared to the control.

### 3.3. Vitamin B6 Deficiency Altered Cecal and Serum Metabolites via Distinctive Pathways

**Figure 4:**
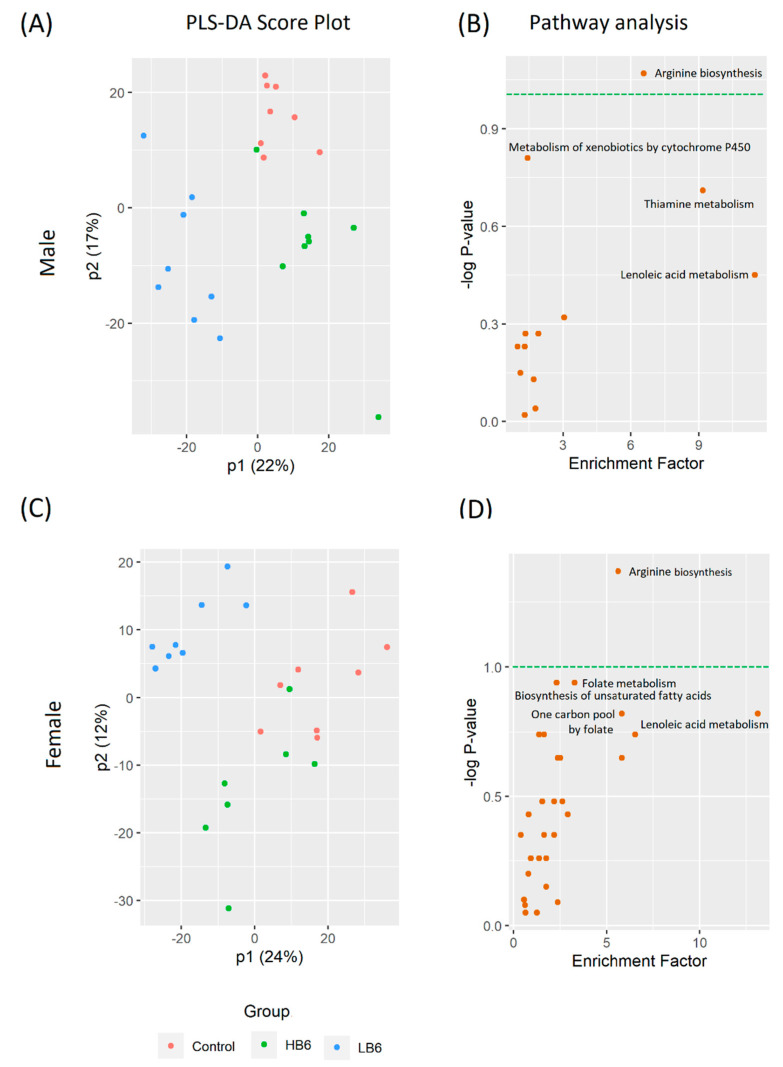
Cecal metabolites that differ in LB6 rats compared to control and HB6 rats (*n* = 8/group). Partial least square-discriminant analysis (PLS-DA) score plot for male (**A**) (R2Y = 0.676, Q2Y = 0.425, *p* < 0.05) and female (**C**) R2Y = 0.671, Q2Y = 0.408, *p* < 0.05) metabolites with their corresponding affected metabolic pathways modulated in LB6 male (**B**) and female (**D**) rats compared to the control. *n* = 7–8/group of each sex.

**Figure 5:**
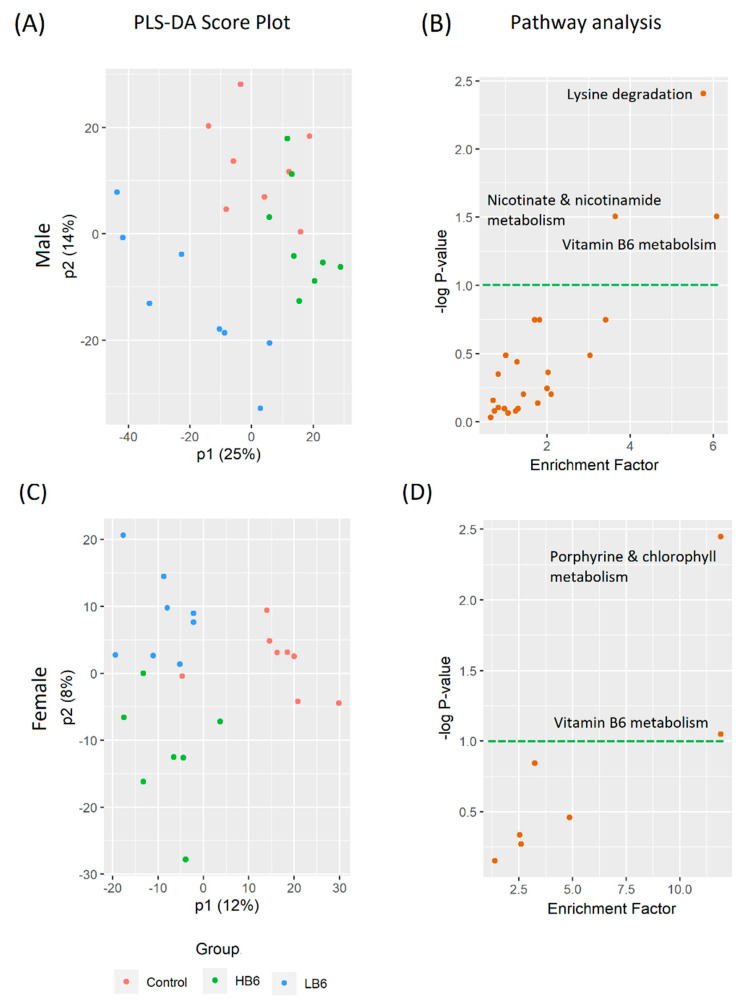
Serum metabolites that differ in LB6 rats compared to control and HB6 rats (*n* = 8/group). Partial least square-discriminant analysis (PLS-DA) score plot for male (**A**) (R2Y = 0.533, Q2Y = 0.276, *p* < 0.05) and female (**C**) (R2Y = 0.674, Q2Y = 0.133, *p* < 0.05) metabolites with their corresponding affected metabolic pathways modulated in LB6 male (**B**) and female (**D**) rats compared to the control. *n* = 7–8/group of each sex.

After peak filtering and blank subtraction, we detected as many as 1590 and 1961 metabolite features in cecal and serum extracts, respectively. Similar to the cecal microbiota, we found a significant sex effect in both the cecal and serum metabolomics analysis (Supplementary Figure S4). A PLS-DA analysis showed significant separation of LB6 cecal metabolites in both male (R2Y = 0.676, Q2Y = 0.425, *p* < 0.05, Figure 4A) and female rats (R2Y = 0.671, Q2Y = 0.408, *p* < 0.05, Figure 4C). Similarly, we also found a significant separation of serum LB6 metabolites from the control and the HB6 rats in both sexes (male: R2Y = 0.533, Q2Y = 0.276, *p* < 0.05, Figure 5A; female: R2Y = 0.674, Q2Y = 0.133, *p* < 0.05, Figure 5C).

**Figure 6:**
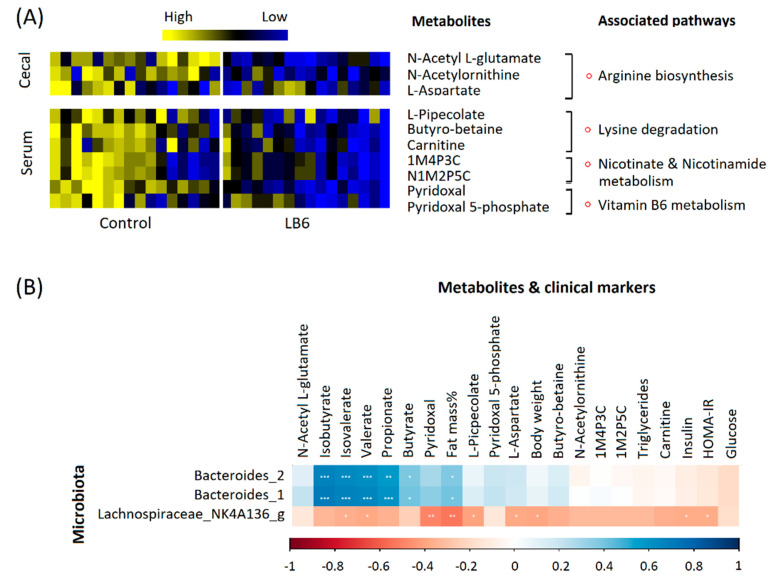
Putatively identified metabolites of significantly altered pathways in LB6 rats compared to control rats (**A**), and Pearson correlation of gut microbiota significant in both sexes with selected metabolites and clinical markers (**B**). 1M4P3C, 1-Methyl-4-pyridone-3-carboximide; N1M2P5C, N1-Methyl-2-pyridone-5-carboxamide. *n* = 7–8/group of each sex.

We further conducted a Mummichog algorithm-based mass spectrometry peak to pathway analysis to identify the pathways affected by vitamin B6 deficiency in the cecal contents, and the host using methods previously described [23]. Interestingly, vitamin B6 deficiency altered gut-associated metabolism (Figure 4B,D) and serum-linked metabolism (Figure 5B,D) in distinctive ways. In the cecal contents, arginine biosynthesis was highly impaired in LB6 rats irrespective of sex, followed by folate and unsaturated fatty acids biosynthesis in female rats. On the other hand, serum metabolites predicted porphyrine and chlorophyll metabolism and vitamin B6 metabolism in female rats, while lysine degradation, nicotinate and nicotinamide metabolism and vitamin B6 metabolism was impaired in male rats in LB6 compared to control. The normalized intensities of the putatively identified metabolites (Supplementary Table S2) of selected pathways associated with the cecal and host-related pathways are shown in Figure 6.

Measurement of cecal short-chain (SCFAs) and branched-chain fatty acids, namely propionate, butyrate, isobutyrate, valerate and isovalerate concentrations, showed significantly reduced concentrations in the cecal matter of LB6 rats in both sexes compared to the control and HB6 rats, while acetate levels did not change (Supplementary Figure S5).

Finally, we conducted a correlation analysis between primary metabolic markers and the gut microbiota that differed significantly in both sexes. *Lachnospiraceae*_NK4A136_group was negatively associated with body weight (r = −0.39, *p* = 0.035) and fat mass ((r = −0.53, *p* = 0.003), while *Bacteroides* were positively associated with percent fat mass ((r = 0.4, *p* < 0.05) and SCFAs and branched-chain fatty acids including isobutyrate (r = 0.70, *p* < 0.001), isovalerate (r = 0.67, *p* < 0.001), valerate (r = 0.65, *p* < 0.001), propionate (r = 0.60, *p* < 0.001) and butyrate (r = 0.40, *p* < 0.05).

## 4. Discussion

**Figure 7:**
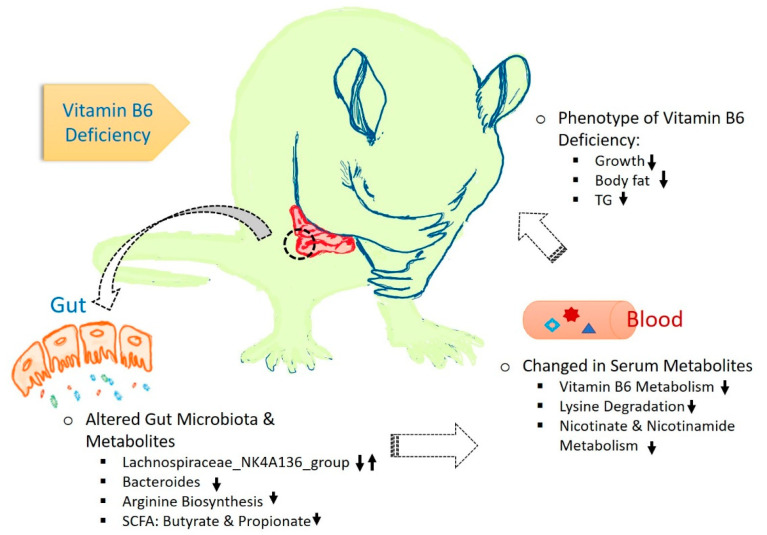
Proposed model showing the interrelationship between the gut microbiota, microbial metabolites and the host metabolism in vitamin B6 deficiency. Up arrows indicate upregulation, down arrows indicate downregulation and double arrows indicate mixed regulation.

B-vitamins, including vitamin B6, play a crucial role in human metabolism due to their involvement as cofactors in a plethora of enzymatic reactions [8]. Host B-vitamin requirements are supplied either by external dietary intake or bacterial biosynthesis. However, the number of gut bacteria that can produce specific B-vitamins is limited [3,24]. For instance, there are only six known bacterial species that can produce vitamin B6 in the gut: *Bacteroides fragilis* and *Prevotella copri* (Bacteroidetes), *Bifidobacterium longum* and *Collinsella aerofaciens* (Actinobacteria) and *Helicobacter pylori* (Proteobacteria) [24]. The host absorbs the majority of the endogenously produced B-vitamins, and therefore, limits its availability to the bacterial non-producers [25]. Although PLP-dependent enzymes account for at least 1.5% of prokaryotic genomes [26], little is known about the relationship between diet-derived vitamin B6 and the gut microbiota. Thus, the current study investigated the potential effects of low or high levels of dietary vitamin B6 on the gut microbial profile and host and microbiota metabolites. We demonstrate that a dietary vitamin B6 deficiency not only impaired the gut microbial profile but also altered cecal and serum metabolites in rats. A proposed model showing the interrelationship between the gut microbiota, microbial metabolites and the host metabolism is displayed in Figure 7.

In the current study, the reduction in bodyweight of LB6 rats was expected, as demonstrated previously [27]. This reduction in bodyweight is putatively explained by lower lipid biosynthesis, as indicated by a lower circulating TG levels and reduced body fat. Williams et al. also reported a reduction in body fat in B6 deficient rats when fed a diet containing either 10 or 20% cottonseed oil [28]. Despite the marked reduction in body fat and serum TG, vitamin B6 deficiency did not affect serum, glucose and insulin and insulin resistance. Vitamin B6 deficiency was found to be associated with the onset of diabetes in individuals with obesity and during pregnancy [29]. However, in the current study, the induction of the deficiency over 6 weeks might not have been long enough to alter serum glucose and insulin levels, or it is possible that the significantly low fat mass of LB6 rats or increased abundance of *Lachnospiraceae* negated such impairments.

According to our knowledge, this is the first time that the direct impact of host dietary vitamin B6 levels on the gut microbial profile has been reported. Unexpectedly, vitamin B6 deficiency resulted in sex-specific alterations to gut microbiota and related metabolites. The reason for this sexual dimorphism in microbiota response to low vitamin B6 intake is not known but could indicate that vitamin B6 is involved in biological functions that differ by sex or that there are unique sex-by-diet interactions that influence microbiota response although these possibilities remain speculative at this time. One of the most intriguing findings of the current study was the increased relative abundance of *Lachnospiraceae*_NK4A136_group and the decrease of *Bacteroides* in LB6 rats. *Lachnospiraceae*_NK4A136_group, a genus of the *Lachnospiraceae* family under the class Clostridia, is a major constituent of the mammalian gastrointestinal tract [30]. The health benefits of microbiota belonging to the *Lachnospiraceae* family have shown mixed responses [31]. For instance, a higher abundance of *Lachnospiraceae* has been associated with improved body weight and insulin sensitivity due to a higher production of butyrate and propionate [32,33], while others suggested a higher abundance is linked to obesity most likely due to higher acetate production [34,35]. The lack of distinction down to the strain level might explain some of these disparate findings as, at this moment, most studies rely on analysis at the family or genus level, which limits our ability to distinguish species or strains of bacteria having different biosynthetic abilities. In the current study, some bacteria belonging to *Lachnospiraceae_*NK4A136*_*group might contribute to the reduction in the growth of LB6 rats as it is negatively associated with body weight and percentage fat. Similarly, *Bacteroides*, another major constituent of mammalian gut microbiota, can act as commensal, mutualist or even pathobiont depending on the microbial composition and the availability of nutrients [36]. Interestingly, colonization with *Bacteroides* corrects immune defects in germ-free mice [37]. A meta-analysis also demonstrated the association of low levels of *Bacteroides* with inflammatory bowel disease [38]. As such, the positive association of this bacteria with serum pyridoxal, cecal butyrate and propionate indicates potential protective functions of *Bacteroides* against metabolic perturbations or immune function in LB6 rats, a novel role of vitamin B6 previously described [11,39].

Pathway analysis revealed that vitamin B6 deficiency substantially altered host cecal and serum metabolites. In the cecum, arginine biosynthesis was markedly impaired in both sexes. Arginine not only acts as a substrate for protein synthesis but also as an important precursor for a variety of molecules linked to cell function, such as nitric oxide [40]. The significant reduction of the intermediate metabolites of arginine biosynthesis, including N-acetylglutamate, N-acetylornithine and L-aspartate, might be due to the downregulation of the PLP-dependent enzymes linked to this pathway such as acetylornithine aminotransferase [41] and aspartate transaminase [42]. The health benefits of arginine supplementation have been well documented including reductions in nitric oxide-linked endothelial dysfunction, improvements in innate and acquired immunity and the inhibition of tumour growth [40]. As the enzymatic pathways of arginine biosynthesis are widely shared across many bacterial genes, it is possible that a significant impairment of bacterial de novo protein synthesis and related cell functions could exist in LB6 rats. With the close link between vitamin B6 and folate metabolism, the impairment in folate biosynthesis in LB6 rats might also be crucial. Around 37% of host folate requirement is believed to be produced by the gut microbiota in humans [43]. Several strains belonging to *Lactobacillus* and *Bifidobacterium* are known to have de novo folate biosynthetic capacity [44]. Bacterial folate biosynthesis requires a broad category of biomolecules characterized by the presence of a complex of pterin, para-aminobenzoate and glutamate subunits [43] whose synthesis requires PLP-dependent enzymes such as 4-amino-4-deoxychorismate lyase [45].

Serum metabolomics analysis detected a defect in vitamin B6 metabolism in LB6 rats, which was entirely expected. Additionally, several other PLP-dependent pathways, including lysine, nicotinate and nicotinamide metabolic reactions, were also impaired due to vitamin B6 deficiency. Lysine is an essential amino acid, and its pipecolate degradation pathway requires a PLP-dependent enzyme called 2-aminoadipate aminotransferase to convert L-2-aminoadipate to 2-oxoadipate [46]. Impairment in lysine degradation has been found to be associated with PLP-dependent epilepsy, which could lead to developmental delays [47]. On the other hand, PLP is indirectly required to synthesize quinolinic acid, the end product of tryptophan and the precursor of nicotinate and nicotinamide metabolism [48]. Our finding is in line with the previous study where Shibata et al., showed lower conversion rate of tryptophan to nicotinamide in vitamin B6- deficient rats [49]. Therefore, low circulating levels of vitamin B6 could be related to these PLP-linked metabolic impairments in LB6 rats.

## 5. Conclusions

The current work demonstrates that the dietary deficiency but not surplus of vitamin B6 alters the gut microbial community and the associated host and microbial metabolites as assessed in serum and cecal contents. Most notably, bacteria-produced vitamin B6 was not sufficient to sustain the metabolism of the gut microbiota and led to severe impairments in several PLP-dependent metabolic reactions with some similar and other distinctive pathways in male and female rats. We report that vitamin B6 deficiency resulted in a marked deterioration in arginine biosynthesis in the gut, and the impairment in these metabolic pathways may have led to the selective growth of certain gut bacteria. Noteworthy reductions in the relative abundance of microbiota belonging to the family *Bacteroidaceae*, including *Bacteroides*, and increases in *Lachnospiraceae* such as *Lachnospiraceae*_NK4A136_group were seen with vitamin B6 deficiency. Overall, this work suggests that micronutrient deficiency, such as vitamin B6 deficiency, might significantly impact host gut microbiota and its metabolites and therefore warrants additional consideration when evaluating the effects of diet on the microbiome.

## Supplementary Materials

Supplementary Materials can be found at https://www.mdpi.com/2227-9059/8/11/469/s1.

## Abbreviations

LB6	Low vitamin B6
HB6	High vitamin B6
PLP	Pyridoxal 5-phosphate
TG	Triglycerides
HOMA-IR	Homeostatic Model Assessment of Insulin Resistance
PCoA	Principal Coordinate analysis
PLS-DA	Partial Least Square Discriminant Analysis
SCFA	Short Chain Fatty Acid
