# Supplementary material for: Dietary Vitamin B6 Deficiency Impairs Gut Microbiota and Host and Microbial Metabolites in Rats
Source: Biomedicines. 2020 Nov 2;8(11):469. doi: 10.3390/biomedicines8110469 (PMC7693528; doi:10.3390/biomedicines8110469)
Supplement: Supplementary file 1 [file biomedicines-08-00469-s001.pdf]

International Journal of
Molecular Sciences

MDPI

Supplementary material

Vitamin B6 deficiency impairs gut microbiota and its metabolites in rats

Shyamchand Mayengbam [1], Faye Chleilat [2] and Raylene A. Reimer [2,3]*

[1] Department of Biochemistry, Memorial University of Newfoundland, St. John's, NL, A1C 5S7; smayengbam@mun.ca
[2] Faculty of Kinesiology, University of Calgary, Calgary, AB T2N 1N4; Fatima.chleilat1@ucalgary.ca
[3] Department of Biochemistry and Molecular Biology, Cumming School of Medicine, University of Calgary, Calgary, AB T2N 1N4; reimer@ucalgary.ca

* Correspondence: Raylene A. Reimer, reimer@ucalgary.ca;

International Journal of
Molecular Sciences

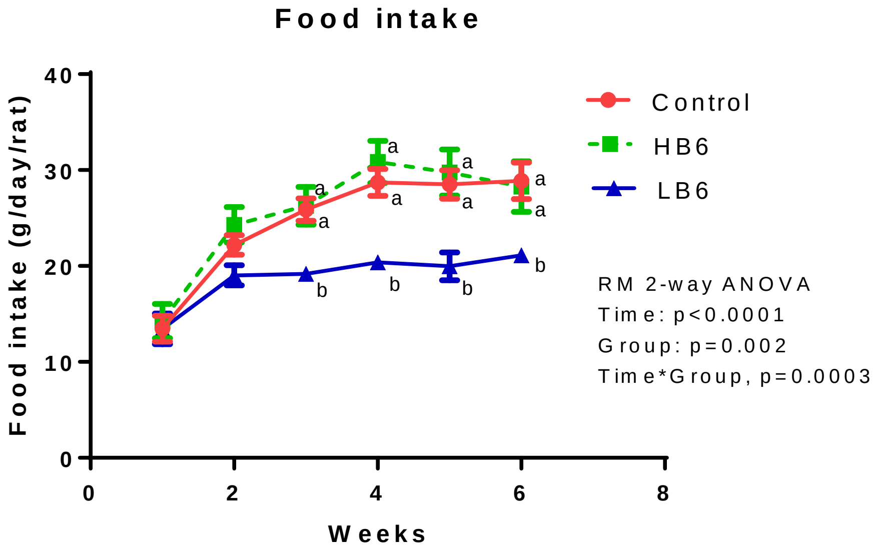

Supplementary Figure 1. Effect of vitamin B6 levels on food intake of rats fed control, HB6 and LB6 for 6 weeks. Values are mean ± SD (n=15-16/group). Values with different superscripts indicate significant differences between groups at the same time points ($p < 0.05$). HB6, high vitamin B6; LB6, low vitamin B6.

International Journal of
Molecular Sciences

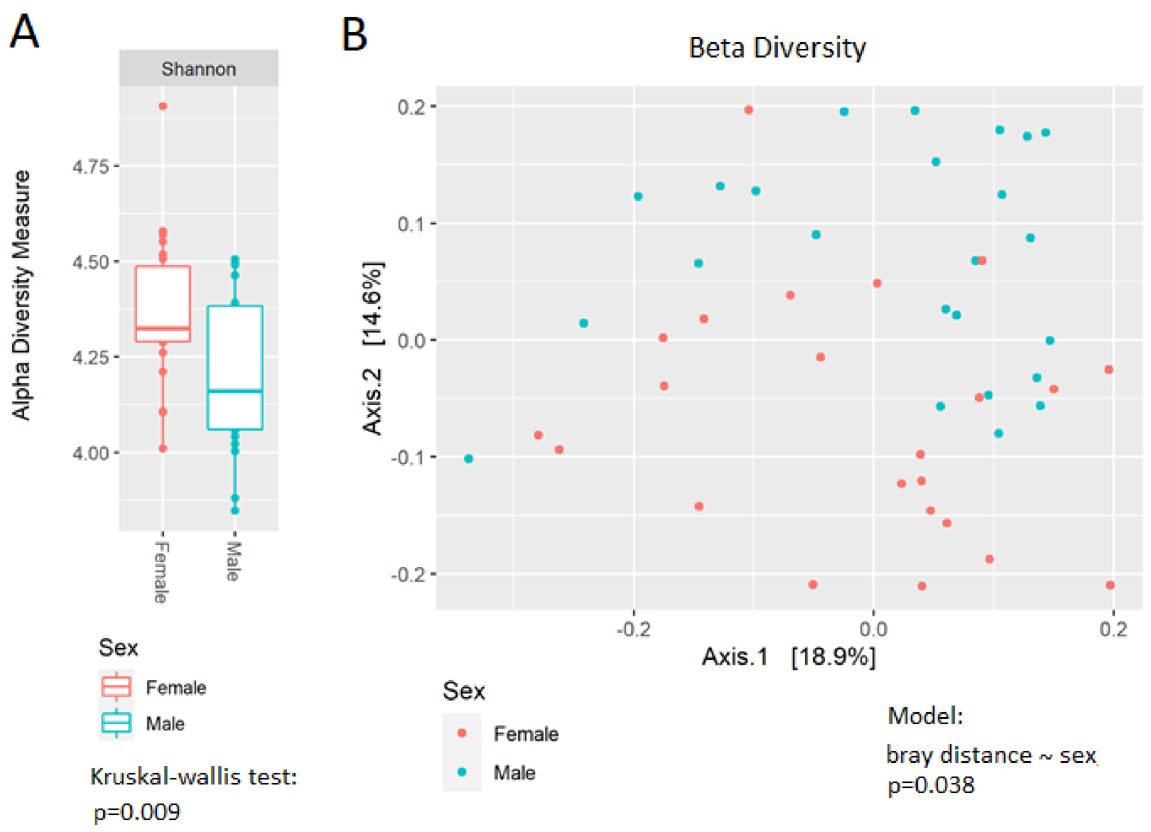

Supplementary Figure 2. Cecal microbiota showing significant sex effect for alpha diversity (Shannon index, p=0.009) and beta diversity (p=0.038). n=7-8/group of each sex.

International Journal of
Molecular Sciences

MDPI

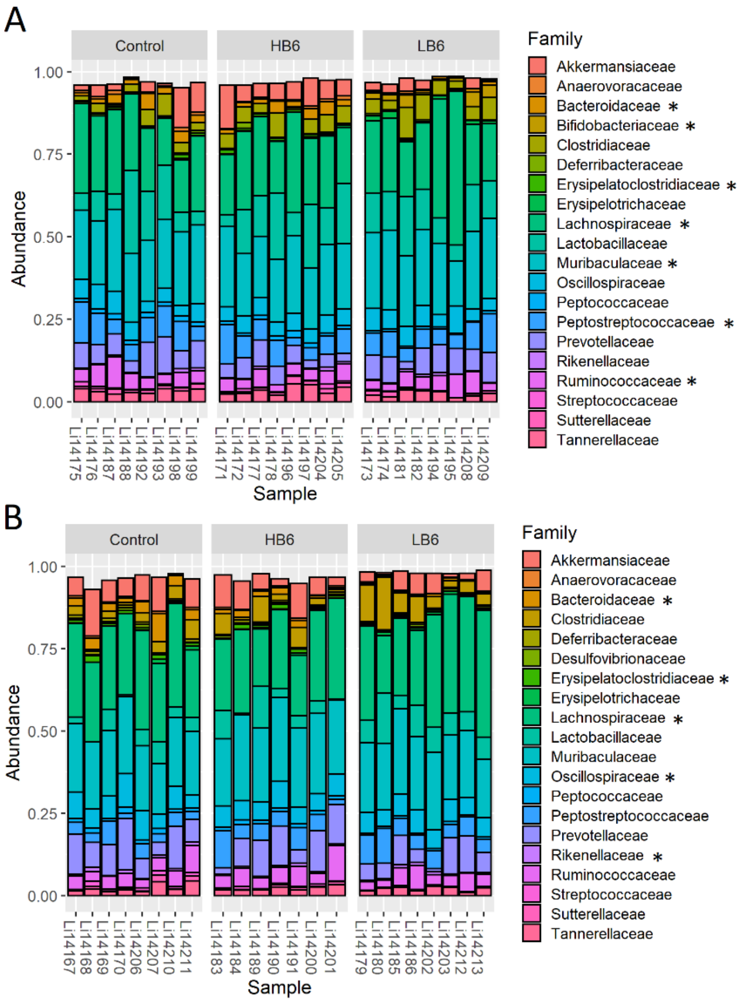

Supplementary Figure 3. The effect of vitamin B6 on the relative abundance of the top 20 family levels for male (A) and female (B) rats. * indicates the taxa that differed between the control and LB6 rats at fdr p-value <0.05 (n=7-8/group of each sex). HB6, high vitamin B6; LB6, low vitamin B6.

International Journal of
Molecular Sciences

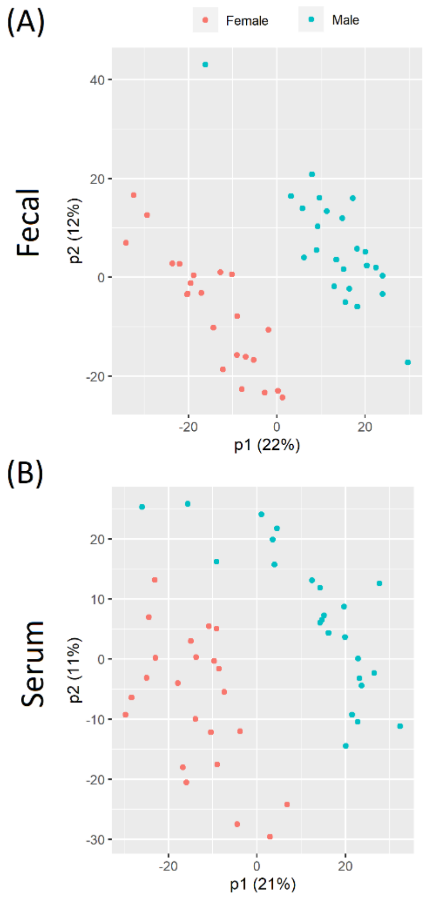

Supplementary Figure 4. PLS-DA score plot for metabolite features showing a significant sex effect on cecal matter (A): R2Y=0.94, Q2Y=0.89, $p<0.05$; and serum (B): R2Y =0.83, Q2Y=0.67, $p<0.05$; n=23-24/group.

International Journal of Molecular Sciences

MDPI

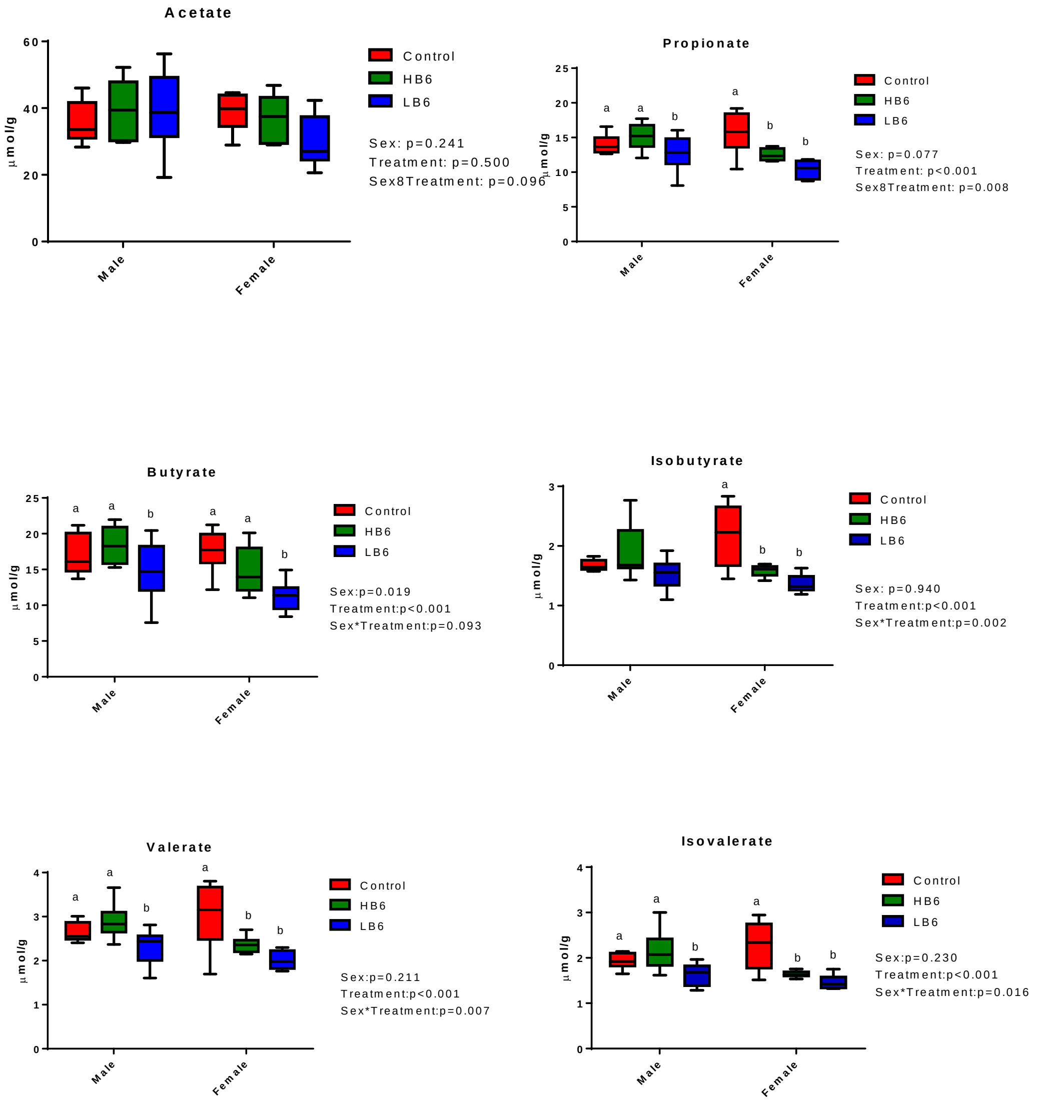

Supplementary Figure 5. Effect of vitamin B6 on cecal short-chain and branched-chain fatty acid concentrations in rats fed control, HB6, LB6 for 6 weeks. Values are mean ± SD (n=7-8/group of each sex). Values with different superscripts indicate a significant difference between the treatment group within sex ($p<0.05$). HB6, high vitamin B6; LB6, low vitamin B6.

International Journal of *Molecular Sciences*

MDPI

Supplementary Table 1. Effect of high and low vitamin B6 on body composition at the end of a 6 week feeding period.

	Male			Female		
	Control	HB6	LB6	Control	HB6	LB6
BMC	12.4±0.7[a]	13.2±1.0[a]	9.7±0.5[b]	9.6±0.3[a]	8.9±0.6[a]	7.8±0.4[b]
BMD	0.16±0.0	0.16±0.0	0.16±0.0	0.16±0.0	0.15±0.01	0.15±0.01
Fat mass	94.9±19.3[a]	110.8±30.5[a]	34.9±4.2[b]	56.8±4.9[a]	46.7±3.6[b]	21.1±3.4[c]
Lean mass	395.5±32.3[a]	422.9±30.2[a]	313.3±30.3[b]	257.5±29.6[a]	259.2±7.2[a]	224.7±11.8[b]
HOMA-IR	4.2±1.4	4.2±2.2	2.3±1.1	1.6±0.4	1.6±0.7	1.1±0.7

Values are mean ± SD (n=7-8/group of each sex). Values with different superscripts indicate significant differences between groups within the same sex ($p<0.05$). BMC, bone mineral content; BMD, bone mineral density; HB6, high vitamin B6; LB6, low vitamin B6.

International Journal of Molecular Sciences

MDPI

Supplementary Table 2. Putatively identified metabolites involved in the significant pathways identified in cecal and serum-linked metabolism with their adducts and mass differences.

Source	mz	KEGG	Adducts	Mass diff	Pathway	Putative metabolites
Cecal	136.040500	C00049	M(S34)+H[1+]	0.000084483770	Arginine biosynthesis	L-Aspartate
	177.104780	C00437	M(Cl37)+H[1+]	0.000139290770	Arginine biosynthesis	N-Acetylornithine
	154.049741	C00624	M-H4O2+H[1+]	0.000058133770	Arginine biosynthesis	N-Acetyl-L-Glutamate
	349.235792	C01595	M+HCOONa[1+]	0.000885565230	Biosynthesis of unsaturated FA	Linoleate
	301.212653	C06427	M+Na[1+]	0.001203370770	Biosynthesis of unsaturated FA	Linolenate
	426.153402	C00415	M-H2O+H[1+]	0.001393594230	Folate biosynthesis	Dihydrofolate
	358.184051	C00504	M-HCOOK+H[1+]	0.001607141770	Folate biosynthesis	Folate
	269.114849	C00921	M-HCOOH+H[1+]	0.000233989230	Folate biosynthesis	Dihydropteroate
Serum	84.080494	C00408	M-HCOOH+H[1+]	0.000361257770	Lysine degradation	L-Pipecolate
	146.117586	C01181	M[1+]	0.000517661000	Lysine degradation	Butyro-betaine
	162.112812	C00487	M+H[1+]	0.000345233230	Lysine degradation	Carnitine
	153.065839	C05843	M+H[1+]	0.000014876770	Nicotinate & nicotinamide metabolism	1-Methyl-4-pyridone-3-carboximide
	175.048303	C05842	M+Na[1+]	0.000449023230	Nicotinate & nicotinamide metabolism	N1-Methyl-2-pyridone-5-carboxamide
	168.065362	C00250	M+H[1+]	0.000157225770	Vitamin B6 metabolism	Pyridoxal
	285.988022	C00018	M+K[1+]	0.000672164230	Vitamin B6 metabolism	Pyridoxal 5 phosphate
